# To Lighten the Burden of Cure: Thyroid Disease in Long-Term Survivors After TBI Conditioning for Paediatric ALL

**DOI:** 10.3389/fped.2021.798974

**Published:** 2022-01-19

**Authors:** Natalia Zubarovskaya, Dorothea Bauer, Leila Ronceray, Ulrike Poetschger, Paulina Kurzmann, Carina Lender, Zoya Kuzmina, Anita Lawitschka

**Affiliations:** ^1^Stem Cell Transplantation Unit, St. Anna Children's Hospital, Medical University Vienna, Vienna, Austria; ^2^St. Anna Children's Cancer Research Institute, Vienna, Austria; ^3^Department of Pediatrics, St. Anna Children's Hospital, Medical University Vienna, Vienna, Austria; ^4^Pulmonology Department, Ottakring Hospital, Vienna, Austria

**Keywords:** hypothyroidism, thyroid cancer, thyroid nodules, total body irradiation, haematopoietic stem cell transplantation, graft-versus-host disease, graft dysfunction

## Abstract

Thyroid disorders are well-studied after allogeneic haematopoietic stem cell transplantation (HSCT) following total body irradiation (TBI)-based conditioning, occurring in 15–30% of paediatric survivors. The toxic effect of TBI is known but data on the role of immunological dysregulation (ID) and chronic graft-versus-host-disease (cGvHD) are scarce. We studied functional and structural thyroid disorders in 97 paediatric ALL patients after TBI-based HSCT, assessing their correlation with patient/transplant characteristics including cGvHD, prolonged immunosuppression and ID. The 10- and 15-year cumulative incidence (CI) of functional disorders was 50 and 60%. Univariate analysis revealed TBI in 6 vs. 8 fractions (*p* = 0.01), an interval between ALL diagnosis and HSCT <1 year (*p* = 0.038), and the application of ATG (*p* = 0.044) as risk factors. The 10- and 15-year CI of structural disorders was 60 and 80%. No correlation between patient/transplant characteristics and structural disorders was observed. cGvHD, prolonged immunosuppression and additional radiotherapy were not associated with any thyroid disease. We observed a significant correlation between ID and the development of thyroid dysfunction in patients with structural changes (10-year CI: 77% for patients with ID vs. 56% without ID, *p* = 0.02). The impact of our results on thyroid follow-up evaluations and the significance of hormonal replacement therapy are discussed.

## Introduction

The endocrine system is commonly affected by high-dose chemotherapy and/or irradiation given prior to allogeneic haematopoietic stem cell transplantation (HSCT) during childhood. Thyroid failure after total body irradiation (TBI) as conditioning for paediatric HSCT is well-studied (1, 2). Known risk factors for thyroid disorders are younger age at HSCT (<9 years) and use of TBI for conditioning (1–4); these have also been correlated with the development of thyroid cancer (2, 5). To reduce transplant-related toxicity, an aim of clinical research in the field of paediatric HSCT has been replacement of TBI with chemoconditioning. However, recently Peters et al. showed significantly higher 2-year event-free survival in paediatric patients with acute lymphoblastic leukaemia (ALL) receiving HSCT after TBI-based conditioning vs. after chemoconditioning (6).

Hypothyroidism is usually a relatively early complication after HSCT but it can manifest at any time point, with an increasing incidence during follow-up (2). The most common functional thyroid disorders after HSCT include compensated and overt hypothyroidism as well as immune thyroiditis. The reported incidence of hypothyroidism varies depending on the duration of follow-up, ranging from 15 to 30% for compensated hypothyroidism and from 10 to 15% for overt hypothyroidism. The incidence of immune thyroiditis has been reported to be much lower, at 0.5% (1–3).

In addition to TBI, thyroid dysfunction is associated with busulfan-based conditioning regimens (2). Recently, Slatter et al. described functional thyroid disorders after HSCT for primary immunodeficiencies in patients who had no history of pre-transplant chemotherapy and who received busulfan-based TBI-free conditioning (7). Besides direct toxic damage by TBI or busulfan, an additional alteration of the thyroid caused by virally induced inflammation (e.g., cytomegalovirus reactivation) or by adoptively transferred, donor-derived thyroid antibodies has been described (8). Isshiki et al. observed a correlation between immune thyroiditis and extensive chronic graft-versus-host disease (cGvHD), with the latter being the main immunological complication after HSCT (9). In the context of immunological damage of thyroid, Savani et al. detected an association between prolonged immunosuppressive treatment (but not cGvHD *per se*) and thyroid alterations (10). A significant association between thyroid autoimmunity and papillary thyroid cancer has been observed in non-HSCT adult patients (11). In children, primary thyroid cancer is a rare event (0.5–3 cases per 1,000,000 patients per year), constituting 1.8–3.0% of all childhood cancers (12–14).

In HSCT patients, Cohen et al. reported from a study on behalf of the European Society of Bone and Marrow Transplantation (EBMT) Late Effects Working Party a significantly higher risk (relative risk, 24.61) of developing thyroid carcinoma in patients transplanted when <10 years old compared with when 11–20 years old (15). Other significant risk factors were irradiation, female sex, and cGvHD (15). Benign and malignant thyroid nodules have been reported in transplanted patients following TBI in paediatric retrospective studies at an incidence of 16 and 8%, respectively (16, 17).

Taking together this published evidence, we hypothesised that thyroid disease after paediatric HSCT is not only caused by toxicity but also by immune dysregulation and cGvHD. To explore this, we conducted a single-centre retrospective study in a paediatric cohort of ALL patients after TBI-based conditioning and HSCT. The aim of the study was to shed light on thyroid disease after HSCT, including functional and structural disorders such as benign and malignant thyroid tumours, and to investigate the potential association between thyroid disease and GvHD, prolonged immunosuppressive treatment and humoral immune dysregulation.

## Materials and Methods

A total of 97 paediatric and adolescent ALL patients who underwent HSCT at the St. Anna Children's Hospital, Vienna, between October 1984 and September 2016 were included in this retrospective study. All patients received TBI-based myeloablative conditioning with 10–12 Gy in 6 or 8 fractions and standard prophylaxis for GvHD, microbial infections and fungal infections according to institutional guidelines. Inclusion criteria included being alive on day +360 after HSCT, no history of thyroid dysfunction prior to HSCT, and no ALL relapse during the observation time. Written informed consent from parents, guardians, and/or patients in accordance with the Declaration of Helsinki and the institutional review board of the Medical University of Vienna and the St. Anna Children's Hospital was obtained.

We collected patient and transplant characteristics, including the use of additional cranial irradiation at any timepoint before HSCT. Acute GvHD (aGvHD) was graded according to the modified Glucksberg criteria (18) and cGvHD was graded according to the 2005 National Institute for Health (NIH) Consensus Criteria (19). Prolonged immunosuppressive treatment was retrieved from patients' medical records. Prolonged immunosuppression was defined being on immunosuppressive therapy 1 year or longer. Humoral immune dysregulation during HSCT follow-up was defined as: (1) the production of non-specific and antithyroid autoantibodies; or (2) levels of immunoglobulins (Ig) such as IgA, IgM, or IgG below or elevated vs. age-adjusted normal values (a list of detected antibodies is presented in the Supplementary Table 1).

Laboratory tests were performed on days +100, +180, +360, and every 6–12 months thereafter following HSCT. Compensated hypothyroidism was defined as elevated levels of thyroid stimulating hormone (TSH; >4.5 mU/L) with normal levels of the thyroid hormones T4 (thyroxine) and T3 (triiodothyronine). Overt hypothyroidism was defined as elevated TSH levels (>10 mU/L) with T4 below age-dependent references. Immune thyroiditis was defined by the occurrence of antithyroid antibodies. During routine aftercare post HSCT at the HSCT Outpatient & Late Effects Clinic of our institution, compensated hypothyroidism is followed up every 3 months and, in the event of increasing TSH levels, hormonal replacement therapy is started.

Ultrasound examination of the thyroid gland was introduced in January 2000 to our routine aftercare programme; it was carried out annually or every 3–6 months in patients with structural thyroid disorders. Ultrasound was performed with a high-resolution transducer of 14.5 MHz supplemented by Doppler US imaging. The ultrasound images were assessed according to patient's age and sex. The standard thyroid imaging reporting and data system (TI-RADS) (20) was used for the evaluation of nodules.

### Statistical Analysis

Cumulative incidences of thyroid disorders were estimated accounting for competing events and compared using Gray's test.

The factors associated with binary outcomes, such as GvHD, prolonged immunosuppressive treatment and humoral immune dysregulation, and the variable thyroid disorders (yes/no) were analysed using univariate binary logistical regression models. Statistical significance was accepted at a *p* < 0.05. All data analyses were performed using SAS 9.4 for Windows.

## Results

### Patient and Transplant Characteristics

We studied thyroid disorders in 97 paediatric long-term survivors of ALL following HSCT with a median follow-up of 7.7 years (range 1.0–21.8 years). Median age at transplantation was 10.2 years (range 2.4–26.2 years). Patient and transplant characteristics are shown in Table 1.

**Table 1:** Patients' baseline characteristics, HSCT characteristics and outcomes during follow-up post HSCT.

**Characteristic**	
Total, *n* (%)	97 (100%)
Female	30 (31%)
Male	67 (69%)
Median age at HSCT, years (interquartile range)	10.3 (2.4–26.2)
Time from ALL diagnosis to HSCT, *n* (%)	
≤1 year	63 (64%)
>1 year	34 (36%)
Remission status at HSCT, *n* (%)	
CR1	73 (75%)
≥CR1	24 (25%)
Number of TBI fractions, *n* (%)	
6	63 (64%)
8	34 (36%)
Additional radiotherapy close thyroid before HSCT, *n* (%)	28 (28%)
Stem cell source, *n* (%)	
Bone marrow	78 (80%)
Peripheral blood stem cells	19 (20%)
Donor type, *n* (%)	
Matched unrelated donor	55 (56%)
Matched sibling donor	42 (44%)
T-cell depletion (anti-thymocyte globulin), *n* (%)	54 (55%)
aGvHD of Grade II–IV, *n* (%)	29 (29%)
cGvHD, *n* (%)	22 (23%)
Mild	3 (14%)
Moderate	2 (9%)
Severe	17 (77%)
Humoral immune dysregulation, *n* (%)	56 (58%)
Antinuclear antibodies (with or without others)	17 (30%)
Thyroid antibodies	10 (18%)
**Outcome**	
Functional thyroid disorders, *n* (%)	39 (40%)
Overt hypothyroidism	8 (20%)
Subclinical hypothyroidism	28 (72%)
Immune thyroiditis	3 (8%)
Structural thyroid disorders (of 61 evaluable patients)^*^, *n* (%)	36 (59%)
Volume changes	23 (64%)
Benign nodules	7 (19%)
Cysts	3 (8%)
Adenoma	1 (3%)
Papillary carcinoma	2 (6%)

* *In 61 patients, results of ultrasound examination were available for analysis of structural thyroid changes (percentages are out of 61). aGvHD, acute graft-versus-host disease; cGvHD, chronic graft-versus-host disease; CR, complete remission; HSCT, haematopoietic stem cell transplantation; TBI, total body irradiation*.

All patients received a TBI-based conditioning regimen with 10–12 Gy in 6 (63/97, 64%) or 8 (34/97, 36%) fractions. Before TBI additional radiotherapy close to thyroid has been applied in 28 patients. About half of all patients (55/97, 57%) received a graft from a matched unrelated donor (MUD) and the other half (42/97; 43%) from a matched sibling donor (MSD). The stem cell source was bone marrow in 78 patients (80%) and peripheral blood stem cells in 19 patients (20%). GvHD prophylaxis comprised cyclosporine A (5 mg/kg) and short-course methotrexate (10 mg/m^2^ on days +1, +3 and +6) in all patients. *In vivo* T-cell depletion with anti-thymocyte globulin (ATG) was applied in 54 of the 55 MUD HSCTs. In the overall cohort, 22 patients (23%) had a history of cGvHD, which was mild in 3 of 22 (14%), and moderate to severe in 19 of 22 (86%). Prolonged immunosuppressive therapy, regardless of cGvHD status, was applied in 23 patients (24%).

Signs of humoral immune dysregulation were evident in 56 (58%) patients (Table 1); 17 of these 56 patients (30%) had a history of cGvHD. In 17 of the 56 patients with immune dysregulation (30%) the expression of antinuclear autoantibodies alone or in combination with other autoantibodies was detected. Ten of the 56 patients (18%) with autoantibodies had anti-thyroid autoantibodies. Of note, the majority (7/10) of patients with anti-thyroid autoantibody expression experienced no signs of thyroid disorder.

### Thyroid Disorders After HSCT During Long-Term Follow-Up

#### Functional Thyroid Disorders

The 10- and 15-year cumulative incidence of functional thyroid disorders was 50 and 60%, respectively. The median interval between TBI application and diagnosis of a functional thyroid disorder (39/97, 40%) was 3.7 years (range 1.0–15.1 years). Of the 39 patients with functional thyroid disorders, 28 had compensated hypothyroidism (72%), eight had overt hypothyroidism (20%) and three had immune thyroiditis (8%).

In the univariate analysis (Table 2), we found a significant correlation between functional thyroid disorders and a short interval (<1 year) between ALL diagnosis and HSCT (*p* = 0.038). Regarding the details of the conditioning regimens, TBI in 6 vs. 8 fractions (*p* = 0.001) and use of *in vivo* T-cell depletion with ATG (*p* = 0.044) were significantly associated with the development of functional thyroid disorders. Of note, additional radiotherapy prior to HSCT and moderate-to-severe cGvHD (vs. none or mild cGvHD) showed a significant negative correlation with functional thyroid disorders (*p* = 0.017 and *p* = 0.047, respectively).

**Table 2:** Correlation between thyroid disorders post TBI-based HSCT with patient and transplant characteristics (univariate analysis and binary logistic regression).

		**Functional thyroid disorders (39 of 97 patients)**			Structural thyroid disorders (36 of 61 evaluable patients^*^)			
**Parameter**		***n*** **(%)**	**10-year cumulative incidence**	***P*** **-value (χ^2^)**	***n*** **(%)**	**10-year cumulative incidence**	***P***-value **(χ^2^)**	
**Univariate analysis**								
Interval between	<1 year	19 (49%)	56%	0.038 (4.318)	16 (45%)	79%	0.985 (0.0003)	
ALL and HSCT	≥1 year	20 (51%)	32%		20 (55%)	77%		
Age at HSCT	<6 years	11 (28%)	63%	0.345 (2.127)	11 (31%)	81%	0.240 (2.784)	
	6–10 years	10 (26%)	26%		10 (28%)	76%		
	≥10 years	18 (46%)	36%		15 (41%)	79%		
Sex	Female	13 (33%)	38%	0.825 (0.04)	10 (28%)	76%	0.476 (0.507)	
	Male	26 (67%)	40%		26 (72%)	83%		
Stem cell source	PBSC	8 (20%)	42%	0.530 (0.394)	7 (19%)	81%	0.511 (0.430)	
	BM	31 (80%)	30%		29 (81%)	77%		
Donor	MSD	13 (33%)	30%	0.08 (2.937)	10 (28%)	78%	0.730 (0.116)	
	MUD	26 (67%)	46%		26 (72%)	79%		
Anti-thymocyte	No	13 (34%)	28%	0.044 (4.037)	9 (25%)	78%	0.730 (0.116)	
globulin	Yes	26 (66%)	49%		27 (75%)	79%		
Number of TBI	6	30 (30%)	56%	0.001 (11.44)	28 (78%)	82%	0.265 (1.230)	
fractions	8	9 (9%)	18%		8 (22%)	62%		
Additional	Yes	6 (6%)	15%	0.017 (5.730)	10 (28%)	84%	0.656 (0.198)	
radiotherapy	No	33 (34%)	48%		26 (72%)	76%		
cGvHD	Moderate or severe	4 (10%)	21%	0.047 (3.945)	6 (17%)	66%	0.2111 (1.559)	
	None or mild	35 (90%)	45%		30 (83%)	80%		
**Parameter**			**Odds ratio**		**95% confidence interval**			***P*** **-value**
**Binary logistic regression**								
cGvHD (any severity)			1.467		0.656–3.279			0.350
cGvHD (moderate/severe vs. none/mild)			0.303		0.092–0.993			0.048
Prolonged immunosuppressive treatment			0.590		0.216–1.610			0.302
Humoral immune dysregulation			2.512		0.832–7.581			0.102

* *In 61 patients, results of ultrasound examination were available for analysis of structural thyroid changes (percentages are out of 61). BM, bone marrow; cGvHD, chronic graft-versus-host disease; HSCT, haematopoietic stem cell transplantation; MUD, matched unrelated donor; MSD, matched sibling donor; PBSC, peripheral blood stem cells; TBI, total body irradiation*.

Additionally, in the binary logistic regression we found a significant negative impact of moderate-to-severe cGvHD (vs. no or mild cGvHD) on the development of functional thyroid disorders (odds ratio, 0.303; 95% confidence interval, 0.092–0.993; *p* = 0.048; Table 2). In the multivariate analysis, only the short interval (≤1 year) between ALL diagnosis and HSCT remained significant (data not shown).

#### Structural Thyroid Disorders

In 61 of 97 patients (63%), results of ultrasound examination were available for analysis of thyroid structure. The cumulative incidence of structural thyroid disorders increased over time, being 50, 60, and 80% after 5, 10, and 15 years, respectively. The median interval between TBI and diagnosis of structural thyroid disorders (36/61, 59%) was 5.6 years (range 1.1–15.0). Of the 36 patients with structural thyroid disorders, sonographic manifestations were volume reduction or enlargement in 23 patients (64%), benign nodules in seven patients (19%), cysts in three patients (8%), adenoma in one patient (3%) and papillary carcinoma (without metastasis) in two patients (6%).

The two patients with papillary carcinoma were aged 5.6 and 13.6 years old at HSCT and 8.0 and 15 years old at papillary carcinoma diagnosis, respectively. Neither patient had a history of GvHD or humoral immune dysregulation but both had a history of thyroid dysfunction (immune thyroiditis in one and compensated hypothyroidism in the other). The latter patient received long-lasting hormonal replacement therapy with levothyroxine. The first signs of benign nodules were detected at 1.7 and 8.0 years after HSCT, respectively; follow-up of thyroid status, including laboratory tests and ultrasonography, was performed every 6 months. Treatment included total thyroidectomy and iodine ablation treatment. Both patients achieved disease-free status with a median follow-up of 6.3 years (6.0 and 6.7 years, respectively).

**Figure 1:**
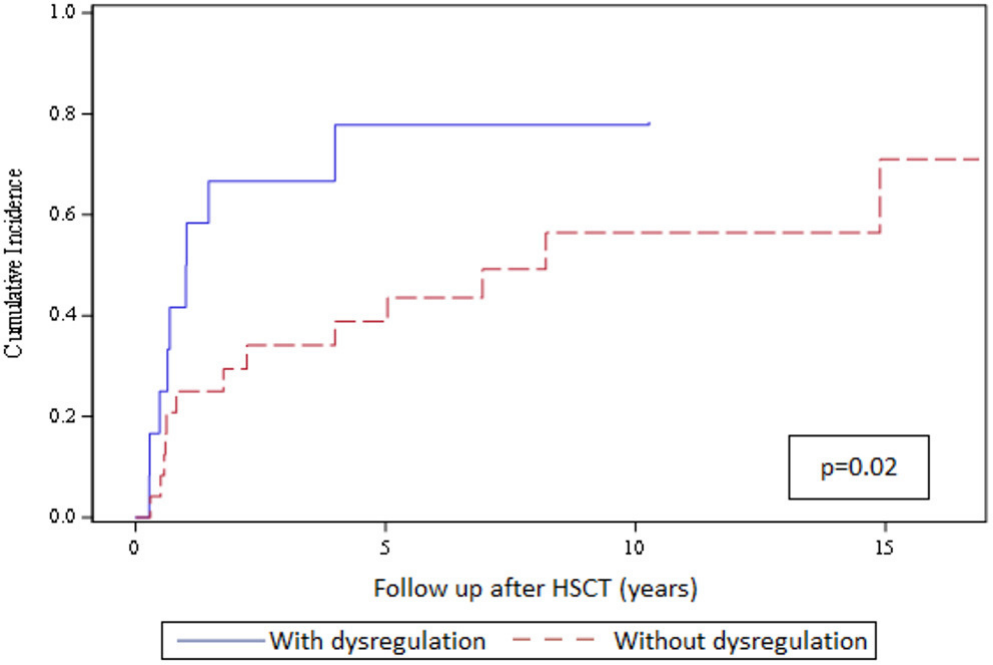
Cumulative incidence of functional thyroid disorders in the 36 patients with structural thyroid changes and with or without signs of humoral immune dysregulation. The top curve represents the cumulative incidence of thyroid functional disorders in patients with structural thyroid changes and immune dysregulation (*n* = 12; blue solid line); the bottom curve represents the cumulative incidence of thyroid functional disorders in patients with structural changes and no immune dysregulation (*n* = 24; red dashed line).

We could not detect any association of structural thyroid disorders with patient and transplant characteristics on univariate analysis (Table 2). Next, we evaluated the impact of humoral immune dysregulation on the cumulative incidence of functional thyroid disorders in the subgroup of 36 patients with structural thyroid changes. Twelve of 36 patients (33%) had signs of humoral immune dysregulation and 24 of 36 (67%) had regular immune reconstitution. The cumulative incidence of functional thyroid disorders at 10 years was significantly higher for patients with vs. without signs of immune dysregulation (77 vs. 56%, respectively; *p* = 0.02) as shown in Figure 1.

## Discussion

Thyroid disorders after paediatric HSCT using TBI-based conditioning have been well-studied over the last decade (2). Recently, Savani et al. reported an association between thyroid disorders and prolonged immunosuppressive treatment (10). In this present retrospective study on a homogenous cohort of paediatric patients with ALL following TBI-based HSCT, we investigated the incidence of both functional and structural thyroid disorders and the association between these and GvHD, prolonged immunosuppressive treatment and humoral immune dysregulation to gain insight into possible immune-mediated damage to the thyroid gland.

In comparison to published data, we found a high cumulative incidence of functional thyroid dysfunction, 50 and 60% at 10 and 15 years post HSCT, respectively (2, 21, 22). We assume that this outcome was related to our use of regular and very long-term follow-up. The occurrence of immune thyroiditis after TBI conditioning was 8% in our cohort, in line with incidence reported in the literature (2, 21, 23). Contrary to the findings of previous studies (1, 3, 24), we did not find a correlation between the incidence of functional thyroid disorders and female sex, younger age at HSCT (≤12 vs. >12 years), and HSCT in second (vs. first) remission of ALL. Like others, we found a significant relationship between the delivery of TBI in fewer than 8 fractions and functional thyroid disorders. It has been previously reported that the use of more highly fractionated TBI resulted in an increased tissue-sparing effect on the thyroid gland in comparison to the use of single or less fractionated TBI (25). It is well-described that the risk of thyroid damage increases in proportion to applied radiation doses (26, 27). Surprisingly, we could not detect an effect of additional radiotherapy pre HSCT on the incidence of functional thyroid disorders.

In our study, the application of ATG as part of the conditioning regimen was significantly associated with functional thyroid disorders. ATG causes complement-dependent T-cell lysis with the release of cytokines and chemokines, leading to a systemic inflammatory response (28) and to a shift toward hypercoagulation with disseminated intravascular coagulation (29). One may speculate that the thyroid gland may be adversely affected by an ATG-induced inflammatory response.

To our knowledge, the augmentation of the occurrence of thyroid dysfunction in patients who were transplanted within the first year after ALL diagnosis has not been described previously. One possible explanation may be that 1 year or below seems a too short a period for complete regeneration of the thyroid gland before HSCT.

In addition to our results regarding functional thyroid disorders, we observed a high cumulative incidence of structural thyroid disorders which increased over time, being 50, 60, and 80% after 5, 10, and 15 years, respectively. The most common structural disorders observed in patients were thyroid volume changes. Vivanco et al. reported in retrospective study of 76 paediatric patients with haematological malignancies treated with TBI an incidence of benign and malignant nodules over 10 years of 16 and 8%, respectively (17). Faraci et al. published an incidence of malignant nodules of 14% in a retrospective study of 42 paediatric patients after autologous and allogeneic HSCT after TBI-based conditioning with 10 years' follow up (16). In our study, the incidence of benign and malignant nodules was 19 and 6 %, respectively. We observed the same occurrence of benign nodules but the incidence of thyroid cancer was lower compared to data in the literature. Only two out of 97 patients developed papillary carcinoma in our cohort. In HSCT patients, Cohen et al. reported from a study on behalf of the EBMT Late Effects Working Party a significantly higher risk (relative risk, 24.61) of developing thyroid carcinoma in patients transplanted when <10 years old vs. 10–20 years old (15). Other significant risk factors for thyroid carcinoma in that study were irradiation, female sex and cGvHD (15). None of patients with thyroid carcinoma had cGvHD. One reason for the low prevalence of thyroid carcinoma in our study vs. other studies might be a protective effect of our practice of starting hormonal replacement therapy early and in the absence of clinical signs of hypothyroidism. In our experience, early introduction of hormonal replacement therapy seems beneficial both with regard to diminishing the risk of thyroid adenoma and carcinoma and to minimising growth failure and delayed development (30, 31).

In our study, presence of a functional thyroid disorder was inversely correlating with cGvHD and was not associated with prolonged immunosuppressive treatment. Indeed, Savani et al. found in adult HSCT patients no correlation between thyroid dysfunction and cGvHD but, in contrast to our data, did find a correlation with prolonged immunosuppression (10). The authors speculated that the thyroid gland might be susceptible to damage by prolonged immunosuppressive treatment directly or to immune-mediated damage. In this regard, we found a significant impact of signs of humoral immune dysregulation on the cumulative incidence of developing functional thyroid disorders in the patient subgroup with structural changes. The cumulative incidence of a thyroid functional disorder at 10 years was significantly higher for patients with vs. without humoral immune dysregulation (77 vs. 56%, respectively; *p* = 0.02). Additionally, Slatter et al. found evidence of immune-mediated thyroid damage in paediatric patients with primary immunodeficiencies after HSCT with chemotherapy-based conditioning regimens (7).

So far, the role of humoral immune dysregulation after HSCT with regards to the expression of specific and non-specific autoantibodies remains unclear; we did not find a higher prevalence of immune thyroiditis in patients expressing antithyroid antibodies than in those patients without antithyroid antibodies. The expression of autoantibodies after HSCT usually reflects an impairment of the transplanted adaptive immune system where immunoregulatory mechanisms are not yet well-established (32).

Our study has potential limitations: it was a retrospective study and not all patients were evaluated by ultrasound. Furthermore, clinical details regarding chronic inflammation and viral complications were not available although they would have been of interest.

In conclusion, in the long-term follow-up of paediatric patients with ALL after HSCT with TBI-based conditioning, we found a high incidence of both functional and structural thyroid disorders, with incidence increasing over time. We learned that thyroid damage after HSCT is multifactorial and it is without a direct impact of cGvHD. However, we found a significant correlation between humoral immune dysregulation and the development of thyroid dysfunction. Therefore, we suggest adding the evaluation of humoral immune dysfunction to the regular thyroid follow-up of patients post HSCT, including laboratory tests and ultrasound examination of the thyroid gland. The early implementation of hormonal replacement therapy as a strategy to prevent thyroid adenoma and carcinoma after paediatric HSCT needs to be proven in a prospective, multicentre study.

## Data Availability Statement

The raw data supporting the conclusions of this article will be made available by the authors, without undue reservation.

## Author Contributions

AL and NZ designed the study. NZ, CL, and DB prepared the study file. UP, PK, and ZK analysed the data. NZ and DB interpreted the results. NZ drafted the manuscript. AL critically reviewed and edited the manuscript. All authors approved the final draft for publication.

## Funding

This study received funding from the St. Anna Children's Cancer Research Institute, Vienna, Austria. The funders were not involved in the study design, collection, analysis, interpretation of data, the writing of this article, or the decision to submit it for publication.

## Conflict of Interest

The authors declare that the research was conducted in the absence of any commercial or financial relationships that could be construed as a potential conflict of interest.

## Publisher's Note

All claims expressed in this article are solely those of the authors and do not necessarily represent those of their affiliated organizations, or those of the publisher, the editors and the reviewers. Any product that may be evaluated in this article, or claim that may be made by its manufacturer, is not guaranteed or endorsed by the publisher.

## Abbreviations

ALL: acute lymphoblastic leukaemia
ATG: anti-thymocyte globulin
aGvHD: acute graft-versus-host disease
cGvHD: chronic graft-versus-host disease
EBMT: european society for bone and marrow transplantation
GvHD: graft-versus-host disease
HSCT: haematopoietic stem cell transplantation
Ig: immunoglobulin
MSD: matched sibling donor
MUD: matched unrelated donor
T3: triiodothyronine
T4: thyroxine
TBI: total body irradiation
TSH: thyroid stimulating hormone.
