# Corrigendum: To Lighten the Burden of Cure: Thyroid Disease in Long-Term Survivors After TBI Conditioning for Paediatric ALL

**DOI:** 10.3389/fped.2022.862455

**Published:** 2022-04-14

**Authors:** Natalia Zubarovskaya, Dorothea Bauer, Leila Ronceray, Ulrike Poetschger, Paulina Kurzmann, Carina Lender, Zoya Kuzmina, Anita Lawitschka

**Affiliations:** ^1^Stem Cell Transplantation Unit, St. Anna Children's Hospital, Medical University Vienna, Vienna, Austria; ^2^St. Anna Children's Cancer Research Institute, Vienna, Austria; ^3^Department of Pediatrics, St. Anna Children's Hospital, Medical University Vienna, Vienna, Austria; ^4^Pulmonology Department, Ottakring Hospital, Vienna, Austria

**Keywords:** hypothyroidism, thyroid cancer, thyroid nodules, total body irradiation, haematopoietic stem cell transplantation, graft-versus-host disease, graft dysfunction

In the original article, there was an error in the **Discussion** section, paragraph ten. “We learned that thyroid damage after HSCT is multifactorial and has a direct impact on rates of cGvHD.” was inaccurately worded resulting in a false representation of the intended message. The corrected paragraph appears below.

“In conclusion, in the long-term follow-up of paediatric patients with ALL after HSCT with TBI-based conditioning, we found a high incidence of both functional and structural thyroid disorders, with incidence increasing over time. We learned that thyroid damage after HSCT is multifactorial and it is without a direct impact of cGvHD. However, we found a significant correlation between humoral immune dysregulation and the development of thyroid dysfunction. Therefore, we suggest adding the evaluation of humoral immune dysfunction to the regular thyroid follow-up of patients post HSCT, including laboratory tests and ultrasound examination of the thyroid gland. The early implementation of hormonal replacement therapy as a strategy to prevent thyroid adenoma and carcinoma after paediatric HSCT needs to be proven in a prospective, multicentre study.”

The authors apologize for this error and state that this does not change the scientific conclusions of the article in any way. The original article has been updated.

## Publisher's Note

All claims expressed in this article are solely those of the authors and do not necessarily represent those of their affiliated organizations, or those of the publisher, the editors and the reviewers. Any product that may be evaluated in this article, or claim that may be made by its manufacturer, is not guaranteed or endorsed by the publisher.

