# Supplementary material for: To Lighten the Burden of Cure: Thyroid Disease in Long-Term Survivors After TBI Conditioning for Paediatric ALL
Source: Front Pediatr. 2022 Jan 19;9:798974. doi: 10.3389/fped.2021.798974 (PMC8809136; doi:10.3389/fped.2021.798974)
Supplement: Supplementary file 1 [file Table_1.pdf]

Supplemental Table 1. Characteristics of autoantibodies analyzed in patients with cGVHD

Name	Acronym	Method of detection	Kit Manufacturer	Reference value	Units
**Antinuclear antibody**	ANA	ELISA	Bio-Rad Laboratories (Hercules, CA, USA)	<1	U
**Extractable nuclear antigen**	ENA	ELISA	Bio-Rad Laboratories	<20	EU
**Anti-mitochondrial**	AMA	ELISA	Bio-Rad	<0.3	U
**Rheumatoid factor**	RF	Immuno nephelometry	Siemens Diagnostics (Tarrytown, NY, USA)	<15	IU/mL
**Liver kidney microsomal**	LK	ELISA	Inova Diagnostics, Inc, San-Diego, USA	<25	U
**Cardiolipin M**	ACA M	ELISA	Inova	<16	MPL
**Cardiolipin G**	ACA G	ELISA	Inova	<12	GPL
**Cyclic citrullinated peptide**	CCP	ELISA	Inova	<20	U
**Double-stranded DNA**	Ds-DNA	ELISA	Farrzyme (Binding Site UK)	<30	IU/mL
**Anti-neutrophil cytoplasmic**	ANCA	Indirect Immuno fluorescence	NOVA Lite ANCA. Inova	neg	U
**Proteinase 3**	Proteinase	ELISA	Quanta Lite (Inova)	<20	U
**Myeloperoxidase**	MP	ELISA	Quanta Lite (Inova)	<20	U
**Parietal**		ELISA	Quanta Lite GPA (Inova)	<25	U
**Centromere**		Immuno fluorescence Assay (IFA)	Zeus Scientific ANA/Hep-2 Cell Culture IFA	<1	U
**SM/Antiribo nucleoprotein**	SM RNP	ELISA	Bio-Rad	<25	EU
**Smith**	SM	ELISA	Bio-Rad	<25	EU
**Sjogren's Syndrome A**	SSA/Ro	ELISA	Bio-Rad	<25	EU
**Sjogren's Sy. B**	SSB/La	ELISA	Bio-Rad	<25	EU
**Smooth muscle**	-	Indirect immune fluorescence	Bio-Rad	neg	
**Thyroglobulin**	-	Chemi luminescence immunoassay	Siemens Diagnostics (Tarrytown, NY, USA)	<40	IU/mL
**Thyroid peroxidase**	-			<35	IU/mL

ELISA = enzyme-linked immunosorbent assay, U = units, EU = ELISA units, IU = international units, mL = milliliter, MPL = IgM phospholipid units, GPL = IgG phospholipid units, neg = negative
